# Role of 3D Printing in Post-op Rehabilitation of Palatal Bone Loss by Mucormycosis: A Survey

**DOI:** 10.7759/cureus.32511

**Published:** 2022-12-14

**Authors:** Anu Gaikwad, Ritumvada Malhotra, Soumendu Bikash Maiti, Amarshree A Shetty, Diya S Rasheed, Laxmikant Kashyap, Ramanpal Singh

**Affiliations:** 1 Department of General Medicine, Dr. D.Y. Patil Medical College, Hospital & Research Centre, Dr. D.Y. Patil Vidyapeeth, Pune, IND; 2 Department of Dentistry, RLC Multispeciality Hospital, Mahasamund, IND; 3 Department of Oral Medicine and Radiology, DJ College of Dental Sciences and Research, Modinagar, IND; 4 Department of Paediatric and Preventive Dentistry, A.B. Shetty Memorial Institute of Dental Sciences, NITTE (Deemed to Be University), Mangalore, IND; 5 Department of General Practice, Rajagiri Hospital, Ernakulam, IND; 6 Department of Periodontology, New Horizon Dental College and Research Institute, Bilaspur, IND; 7 Department of Oral Medicine and Radiology, New Horizon Dental College And Research Institute, Chhattisgarh, IND

**Keywords:** 3d printing, palatal bone, mucormycosis, bone rehabilitation, bone reconstruction

## Abstract

Background: Three dimensions (3D) modeling, printing, and manufacturing can help in personalized and customized surgical reconstruction of complex defects in the craniofacial region with precision by manipulating tissues based on the preoperative assessment, planning the shape of metal and alloplastic materials, and reduction in the total cost and time of the surgery.

Aim: The present survey study aimed to assess the approach of treating surgeons towards the role of 3D printing in post-op rehabilitation of palatal bone loss by mucormycosis.

Methods: One thousand eyes nose and throat (ENT) and maxillofacial surgeons were given a pre-formed structured survey questionnaire to be filled by subjects themselves for their view on the role of 3D printing for rehabilitation and reconstruction of palatal bone loss due to mucormycosis.

Results: Efficacy of 3D printing to print the pneumatic sinus design and palatal contour helping to design accurate support with a lightweight prosthesis, 67.2% (n=672) subjects whereas, exact duplication of the excised tissue, 85.4% (n=854) subjects, to detect and duplicate undercuts, 58.4% (n=584) subjects, 3D printing can be helpful as the proper extension of impression 73.2% (n=732) subjects responded positively. For reconstruction of a lost palate by prosthesis 91.2% (n=912) of study participants, in making obturators using Titanium framework and Polyetheretherketone (PEEK) was given a positive response by 82.2% (n=822) subjects, to fabricate prosthesis obturator required in palatal perforation in case of mucormycosis was given a positive response by 88.1% (n=881) subjects, the role of 3D printing to overlay zygomatic implant prosthesis was responded positively by 68.9% (n=689) study subjects.

Conclusion: The present survey study concludes that 3D printing is a reliable and accurate method for palatal reconstruction following bone destruction by mucormycosis as reported by the majority of ENT and maxillofacial surgeons.

## Introduction

Apart from various surgical modalities for the reconstruction of maxillofacial bones, the most widely used technique is autologous bone grafting. However, autologous bone grafting is associated with disadvantages including donor site morbidity after bone harvesting from the unaffected and healthy area. These shortcomings are compensated with the introduction of three-dimensional (3D) printing which also eliminated the challenges of shaping and harvesting the implant and grafts [1]. Also, various attempts have been done for doing bone reconstruction using 3D printing techniques along with the fabrication of customized artificial medical implants. The implant materials used in bone construction commonly are not degradable which further increases the risk of implant protrusion, infection, and inflammation. To overcome these limitations, recent work is focused to perform reconstruction or regeneration of bone defects utilizing the 3D print scaffolds composed of clinically safe and biodegradable polymers [2].

3D modeling, printing, and manufacturing can help in personalized and customized surgical reconstruction of complex defects in the craniofacial region with precision by manipulating tissues based on the preoperative assessment, planning the shape of metal and alloplastic materials, and reduction in the total cost and time of the surgery [3]. Also, these tools aid in positioning and shaping newly incorporated tissues with precision resulting in improved functional and esthetic outcomes following the reconstructive therapies which further are beneficial for educating the patients. The commonly and frequently used tools in reconstruction therapy including cutting guide placement are made with fused deposition modeling (FDM) or stereolithography (SLA) made of bioinert and sterile materials including polypropylene, poly(methylmethacrylate) (PMMA), or acrylonitrile butadiene styrene (ABS). Implants used usually require additional compatibility in the long term along with mechanical strength. These implants are often sintered from bioglass or titanium [4,5].

Reconstruction of the palatal defects following mucormycosis can be challenging owing to the complexity of the anatomical structures of the palate and various tissue-specific requirements. The 3D printing technologies help scientists, engineers, and clinicians with the ability to get specific solutions for subjects with palatal defects encountered following mucormycosis. Currently, to restore the function and appearance of the palatal morphology, there are three key components including regeneration, reconstruction, and rehabilitation. 3D printing can be used in the rehabilitation process for the fabrication of prostheses to cover and replace damaged palatal tissues [6].

Plastic surgery is used in the reconstruction process using 3D printing techniques to fabricate the custom guides, implanted medical devices, practice models, and fixation devices to get better outcomes in palatal defect reconstruction for mucormycosis, whereas regeneration utilizes the biologic materials for graft replacement. 3D printing can help to get scaffolds or cellular constructs which further send signals to cells that form tissues for regeneration of the bone defects. With the help of these three approaches, 3D printing techniques give chance to make personalized and customized treatment plans and design the solution for improved functional and esthetic outcomes for subjects having palatal bone defects secondary to mucormycosis [7]. The present survey study aimed to assess the approach of treating surgeons towards the role of 3D printing in post-op rehabilitation of palatal bone loss by mucormycosis.

## Materials and methods

The present survey study aimed to assess the approach of treating surgeons towards the role of 3D printing in post-op rehabilitation of palatal bone loss by mucormycosis. The study was done at Dr. D.Y. Patil Vidyapeeth, Pune, Maharashtra, India, after clearance was provided by the Ethical committee from the institute (No: DYPMC/2021/301). The study included 1,000 surgeons primarily involved in the reconstruction of the palatal defects after palatal bone loss from mucormycosis.

The study included participants of both genders from the specialty of eyes, nose and throat (ENT) and oral and maxillofacial surgery who performed the surgery for palatal bone defects reconstructions caused secondary to the mucormycosis and were practicing in either private or public practice. After explaining the detailed study design, informed consent in both written and verbal format was taken from all the responders.

The inclusion criteria for the study were subjects who agreed to participate in the survey, were registered surgeons from either ENT or maxillofacial surgery practice, gave consent, had experience using 3D printing technology and they have been certified as people working with 3D printing. The exclusion criteria were subjects not willing to respond, gave incompletely filled survey forms, were practicing conventional surgery without any 3D printing technique, and did not sign the consent.

**Figure 1:**
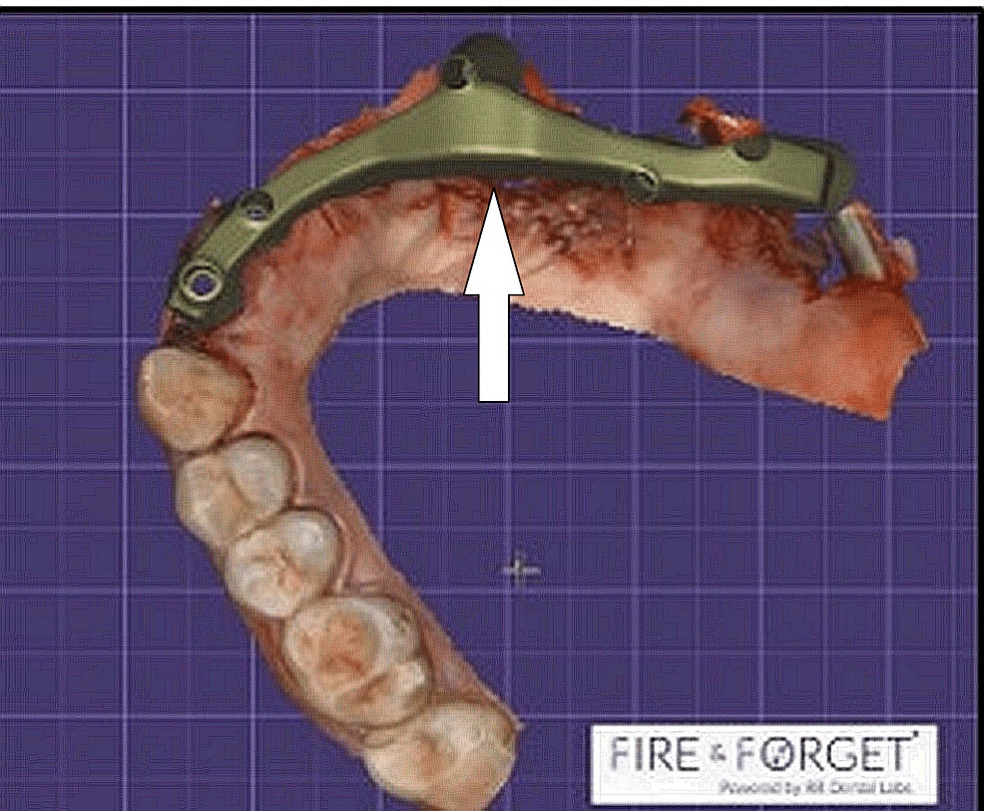
Occlusal rehabilitation using 3D printing following tooth loss by mucormycosis white arrow: 3D printed framework for rehabilitation

After the final recruitment of the responders, they were given a pre-formed structured survey questionnaire to be filled out by the subjects themselves. The identity of the responders was kept anonymous to avoid any bias. The survey questionnaire was formed to assess the approach and experience of the surgeons towards 3D printing concerning printing the sinus and palatal contour, designing prosthesis of lightweight, exact replication of lost palatal tissues and undercuts, making correct size prosthesis, fabricating obturators, facial contours, replacement of lost palatal prosthesis, to aid in deglutition, prevent regurgitation, and as an aid in placing the zygomatic implants to replace missing teeth, and others which are the frequently asked questions (FAQs) while utilizing the 3D prints in palatal reconstruction for bone loss caused secondary to mucormycosis (Figure 1).

The responders were asked to fill out a survey questionnaire on the spot and were collected and analyzed (Table 1).

**Table 1:** Questionnaire used in the survey

Questionnaire
Question 1: Role of 3D print in the pneumatic sinus design and palatal contour helping to design accurate support with lightweight prosthesis
Yes
No
Question 2: Exact duplication of the excised tissue
Yes
No
Question 3: For undercuts that can be detected and duplicated in 3D printing
Yes
No
Question 4: A properly extended impression is not possible in every case
Yes
No
Question 5: Subperiosteal implants can be used
Yes
No
Question 6: For reconstruction of a lost palate by prosthesis so a better correct-sized implant or prosthesis will help in better voice and deglutition, better correction of lost facial features and to prevent regurgitation of food/fluids
Yes
No
Question 7: For making obturators using the Ti framework and PEEK
Yes
No
Question 8: To fabricate a prosthesis like an obturator required in palatal perforation in case of mucormycosis
Yes
No
Question 9: To design the maxillary bone in the palate after a CT scan reading
Yes
No
Question 10: To overlay zygomatic implant prosthesis
Yes
No

The data gathered were analyzed statistically. The results were expressed in numbers and percentages and means and standard deviations and results were formulated.

## Results

The study included 1000 surgeons primarily involved in the reconstruction of the palatal defects after palatal bone loss from mucormycosis. The study included participants of both genders from the specialty of ENT and oral and maxillofacial surgery who performed the surgery for palatal bone defect reconstructions caused secondary to the mucormycosis. The demographics of the responders are described in Table 1. Among included 1,000 participants, 48.7% (n=487) subjects were from the specialty of ENT, whereas the remaining 51.3% (n=513) subjects were oral and maxillofacial surgeons. The mean age of the study subjects was 38.4±6.22 years and was within the age range of 30-66 years. The majority of the study subjects were in the age range of 31-40 years with 36.7% (n=367) subjects followed by 32.4% (n=324) subjects from 41-50 years of age, 23.8% (n=238) subjects from < 30 years of age, and least, 7.1% (n=71) subjects were in the age range of >50 years. There were 63.4% (n=634) male responders and 36.6% (n=366) female participants in the present study. Concerning the years in practice, most of the responders were practicing for 5-10 years with 42.1% (n=421) subjects followed by 24.6% (n=246) subjects with <5 years of practice, 21.3% (n=213) subjects with 10-15 years of practice, and least 12% (n=120) subjects with >15 years of the practice. For the type of practice, 57.8% (n=578) subjects were in private practice and 42.2% (n=422) subjects were from the public sector practice, respectively, as summarized in Table 2.

**Table 2:** Demographics of the study responders

Characteristics	Subgroup	%	n=1000
Mean age (years)		38.4±6.22	
Age range (years)		30-66	
	<30	23.8	238
	31-40	36.7	367
	41-50	32.4	324
	>50	7.1	71
Gender	Males	63.4	634
	Females	36.6	366
Specialty	ENT	48.7	487
	Maxillofacial surgery	51.3	513
Years in practice	<5	24.6	246
	5-10	42.1	421
	10-15	21.3	213
	>15	12	120
Type of practice	Public	42.2	422
	Private	57.8	578

For the FAQs, concerning the efficacy of 3D printing to print the pneumatic sinus design and palatal contour helping to design accurate support with a lightweight prosthesis, 67.2% (n=672) subjects responded positively, whereas 32.8% (n=328) subjects gave a negative response. For the role of 3D printing in the exact duplication of the excised tissue, 85.4% (n=854) subjects responded positively and 14.6% (n=146) subjects responded negatively. To detect and duplicate undercuts, 58.4% (n=584) subjects gave positive responses and 41.6% (n=416) subjects gave negative response.3D printing can be helpful as the proper extension of impression is not possible in every case positively responded by 73.2% (n=732) subjects and negatively by 26.8% (n=268) subjects. To the question that subperiosteal implants can be used, 34.7% (n=347) subjects gave a positive response, whereas 65.3% (n=653) subjects gave a negative response (Table 3).

**Table 3:** Response of responders to frequently asked questions about 3D printing FAQs: frequently asked questions

FAQs	Response	
	Positive n(%)	Negative n(%)
To print the pneumatic sinus design and palatal contour helping to design accurate support with lightweight prosthesis	672 (67.2)	328 (32.8)
Exact duplication of the excised tissue	854 (85.4)	146 (14.6)
For undercuts that can be detected and duplicated in 3D printing	584 (58.4)	416 (41.6)
A properly extended impression is not possible in every case	732 (73.2)	268 (26.8)
Subperiosteal implants can be used	347 (34.7)	653 (65.3)
For reconstruction of a lost palate by prosthesis so a better correct-sized implant or prosthesis will help in better voice and deglutition, better correction of lost facial features and to prevent regurgitation of food/fluids	912 (91.2)	88 (8.8)
For making obturators using the Ti framework and PEEK	822 (82.2)	178 (17.8)
To fabricate a prosthesis like an obturator required in palatal perforation in case of mucormycosis	881 (88.1)	119 (11.9)
To design the maxillary bone in the palate after a CT scan reading	689 (68.9)	311 (31.1)
To overlay zygomatic implant prosthesis	634 (63.4)	366 (36.6)

Other questions asked were the role of 3D printing for the reconstruction of a lost palate by prosthesis so a better correct sized implant or prosthesis will help in better voice and deglutition, better correction of lost facial features and to prevent regurgitation of food/fluids got a maximum positive response by 91.2% (n=912) study participants and negative by only 8.8% (n=88) study subjects. In making obturators using Titanium framework and PEEK was given a positive response by 82.2% (n=822) study subjects and a negative response by 17.8% (n=178) study subjects, to fabricate prosthesis like obturator required in palatal perforation in case of mucormycosis was given positive response by 88.1% (n=881) subjects and negative response by 11.9% (n=119) study subjects. For designing the maxillary bone in the palate after CT scans reading, the response was positive in 68.9% (n=689) subjects and negative in 31.1% (n=311) study subjects. The role of 3D printing to overlay zygomatic implant prosthesis was responded positively by 68.9% (n=689) study subjects and negative by 36.6% (n=366) study subjects as described in Table 3.

## Discussion

The facial area largely governs the esthetics of a person and is usually affected by facial deformities, congenital anomalies, tumors, trauma, and other diseases where mucormycosis constitute a primary etiologic factor in causing the loss of palatal bone architecture and structure further causing regurgitation of fluids, difficulty in mastication, deglutition, and speech as reported by Oh et al. [8] in 2018 and Van-Der-Meer [9] in 2016. In such situations, surgical reconstruction remains the gold-standard treatment modality. However, owing to the functional and anatomic complexity of the soft and hard tissues of the palate and profuse innervation by various nerves and blood vessels, palatal reconstruction is a challenging process which was also suggested by the studies of Lei et al. [10] in 2019 and Wang et al. [11] in 2017.

The use of 3D printing in oral and maxillofacial surgery is a decade-old process. However, their use was limited initially. With the advancement along with the evolution of the accessibility and technologies and low-cost of 3D printers, their use has recently bloomed in maxillofacial surgery depicted by Emodi et al. [12] in 2017 and Guth et al. [13] in 2017. Conventional reconstructive procedures were associated with donor site morbidity from autologous graft harvesting sites such as osteocutaneous flaps and FFF (free fibula graft) for surgical correction of the defects. These techniques showed success and efficacy, however, remodeling is the vital step in bone reconstruction needing precise planning and techniques in palatal reconstruction to maintain the function, structure, and anatomy of the palate. Conventional techniques were associated with these errors along with other complications such as abscess, cellulitis, dehiscence, and/or graft loss as reported by Fang et al. [14] in 2014.

Many oral surgeons nowadays use 3D printing techniques and virtual planning to get better treatment outcomes and better care to provide acceptable outcomes and comfort to their patients. As per the recent analysis in 2017 by Jacobs et al. [15] 3D printing in craniofacial surgery can be used to make contour models, surgical guides, splints, and/or implants where positive space model creation is the most common process involving object printing depending on imaging and external anatomy of the subjects along with being timesaving and emergency process in conditions like fracture and mucormycosis. These findings were in agreement with the present study concerning the efficacy of 3D printing to print the pneumatic sinus design and palatal contour helping to design accurate support with a lightweight prosthesis, 67.2% (n=672) subjects responded positively, whereas 32.8% (n=328) subjects gave a negative response.

Also, 3D printing aids in developing accurate reconstruction plates and surgical guides in subjects with autologous bone grafts as the first treatment choice in replacing the lost structure as reported by Largo [16] in 2018. This was similar to the present study where for the role of 3D printing in the exact duplication of the excised tissue, 85.4% (n=854) subjects responded positively and 14.6% (n=146) subjects responded negatively. Splints are used in orthognathic surgeries for occlusion correction and jaw alignment and to create a negative space, obturators are used in the palatal bone reconstruction to avoid the fluid regurgitation and to plan future orientation and position of teeth and bone and accurate results as described by the previous studies of Ganry et al. [17] in 2017 and Lin et al. [18] in 2020 supporting the present survey was to the role of 3D printing to fabricate prosthesis like obturator required in palatal perforation in case of mucormycosis was given positive response by 88.1% (n=881) subjects and negative response by 11.9% (n=119) study subjects. For designing the maxillary bone in the palate after CT scans reading, the response was positive in 68.9% (n=689) subjects and negative in 31.1% (n=311) study subjects.

Also, 3D printing is highly efficacious in implant planning as it is highly specific in biological, functional, and structural aspects as reported by the systematic review of Louvrier et al. [19] in 2017 that 3D printing is a reliable implant guide which was consistently with the results of the present study where for the role of 3D printing in subperiosteal implants, 34.7% (n=347) subjects gave a positive response, whereas 65.3% (n=653) subjects gave a negative response and for the role of 3D printing to overlay zygomatic implant prosthesis was responded positively by 68.9% (n=689) study subjects and negative by 36.6% (n=366) study subjects.

The limitations of the study were the use of different testing methods, 3D printers, different work applications, and workflows which further made the difficult choice to compare the responses of the different responders. The reliability is also questionable as different surgeons were from two different specialties and work backgrounds.

## Conclusions

With its limitations, the present survey study concludes that 3D printing is a reliable and accurate method for palatal reconstruction following bone destruction by mucormycosis as reported by the majority of ENT and maxillofacial surgeons. Mucormycosis being a fatal fungal infection need early diagnosis and treatment with surgical rehabilitation in the palatal structure needing accurate knowledge about the disease, anatomy, implants, and needed prosthesis.
